# Replicative Study of the Impacts of Applied Behavior Analysis on Target Behaviors in Individuals With Autism Using Repeated Measures

**DOI:** 10.7759/cureus.56226

**Published:** 2024-03-15

**Authors:** Tami Peterson, Jessica Dodson, Frederick Strale,

**Affiliations:** 1 Hyperbaric Oxygen Therapy, The Oxford Center, Brighton, USA; 2 Applied Behavior Analysis, The Oxford Center, Brighton, USA; 3 Biostatistics, The Oxford Center, Brighton, USA

**Keywords:** applied behavioral analysis (aba), naturalistic environment training, mass trials, discrete trial training, repeated measures design, autism spectrum disorder (asd)

## Abstract

Background

The effectiveness of interventions based on applied behavior analysis (ABA) for individuals with autism has been well documented in numerous meta-analyses, systematic reviews, and cost-benefit analyses. However, an observed ‘efficacy-effectiveness gap’ exists, which can be attributed to various factors. This third replication study, therefore, has significant implications for the field. By assessing the impact of ABA treatment, specifically involving discrete trial training and mass trials, within a naturalistic environment, the study provides valuable insights that can inform and improve the delivery of ABA treatments in real-world settings.

Methods

The study was conducted using a repeated measures research design. Retrospective chart review data were collected from 62 individuals with autism, age (M=8.65, SD=4.53), all of whom were level two autistic and required moderate support in communication, socialization, and daily life. These individuals received ABA treatment over five months. The study measured cumulative target behaviors using a repeated measures design, which allowed for the identification of statistically significant differences across 12 time points. This robust methodology ensures the validity and reliability of the study's findings.

Results

Mixed repeated measures analysis of variance (ANOVA) indicated statistical significance (sphericity assumed), F(11,495) = 55.432, p < 0.001 (time). Multiple comparisons using bootstrapped paired t-tests showed p < 0.05 on time points 1-8 and non-significance (p > 0.05) on time points 9-12. There was a significant interaction effect (sphericity assumed) with time x (age category), F(44,495) = 2.338, p < 0.001. Interaction contrasts indicated statistically significant differences over time, mainly within the one-year to four-year-old, five to eight-year-old, and most in the nine to 12-year-old age groups. There was some significance within the 13- to 16-year-old age group and no significance within the 17- to 26-year-old age group.

Conclusions

Over five months, individuals with autism who underwent ABA treatments demonstrated a statistically significant enhancement in general target behaviors. This finding is crucial as it underscores the effectiveness of ABA treatments in a naturalistic environment. Moreover, the study's discovery of a significant interaction between time and age in these behaviors provides valuable insights into the impact of age on treatment outcomes. Extensive large-N studies of general ABA broad effectiveness and repeated measures designs are lacking and can lead to further research to improve quality and outcomes. These findings contribute to the body of empirical evidence and emphasize the importance of replicative efficacy studies in ensuring the reliability of research findings.

## Introduction

Background 

Prevalence

Approximately one in 36 children has been identified with autism spectrum disorder (ASD), according to estimates on ASD prevalence from the Center for Disease Control's (CDC) Autism and Developmental Disabilities Monitoring (ADDM) Network. Autism spectrum disorder is reported to occur in all racial, ethnic, and socioeconomic groups and is nearly four times more common among boys than among girls [1]. Overall, ASD prevalence was lower among non-Hispanic White children (24.3) and children of two or more races (22.9) than among non-Hispanic Black or African American, Hispanic, and non-Hispanic Asian or Pacific Islander children (29.3, 31.6, and 33.4, respectively). The prevalence of ASD among non-Hispanic American Indian or Alaska Native children (26.5) was like that of other racial and ethnic groups. About one in six (17%) children aged between three and 17 years were diagnosed with a developmental disability, as reported by parents, during a study period of 2009-2017. These included autism, attention-deficit/hyperactivity disorder, blindness, and cerebral palsy, among others [1]. 

Efficacy Studies Delineating Applied Behavior Analysis (ABA) Efficacy

Applied behavior analysis therapy has been extensively recognized as the gold standard for the treatment of ASD. This acknowledgment comes from decades of research and a large body of supporting evidence. Yu et al. [2] reported via their meta-analyses containing 14 randomized control trials of 555 participants on the positive impacts of ABA with moderate to high effect sizes, bringing significant benefits for children with ASD. 

Makrygianni et al. [3] meta-analyzed 29 studies and found that ABA programs are moderate to highly effective, bringing significant benefits to children with ASD. Dixon et al. [4], in their randomized controlled trial assessing 28 children with autism, found that the highest intelligence score changes were shown for participants in the comprehensive ABA group. 

Rodgers et al. [5] evaluated 25 studies in a systematic review and meta-analysis of 20 studies to evaluate the clinical effectiveness of an early intensive applied behavior analysis-based intervention for autistic children. They found substantial heterogeneity, and effects varied considerably across studies. They commented that the impact of the intervention on autism symptom severity, language development, and school placement remains uncertain because of limited data. The long-term effects are unclear owing to a lack of follow-up data. 

Further studies into the effectiveness of early intensive applied behavior analysis-based interventions may be warranted if they include well-defined, alternative interventions as comparators and collect relevant outcomes. Consideration should be given to future studies that not only address whether early intensive applied behavior analysis-based interventions are clinically effective but also aim to identify which components of early intensive applied behavior analysis-based interventions might drive effectiveness [5]. 

Eckes et al. assessed the effects of ABA on developmental outcomes in children with ASD and parental stress based on 11 studies with 632 participants. Compared to treatment as usual, minimal or no treatment, comprehensive ABA-based interventions showed medium effects for intellectual functioning and adaptive behavior. Language abilities, symptom severity, or parental stress did not improve beyond the improvement in control groups [6]. 

Gitimoghaddam et al. [7] searched seven online databases and identified systematic reviews for published peer-reviewed English-language studies examining the impact of ABA on health outcomes. They classified measured ABA outcomes into eight categories: cognitive, language, social/communication, problem behavior, adaptive behavior, emotional, autism symptoms, and quality of life. Anderson & Carr [8] highlighted numerous meta-analyses, systematic reviews, and cost-benefit analyses that testified to the effectiveness of interventions based on ABA with autistic individuals. However, there is a noted “efficacy-effectiveness gap” due to factors such as individuals’ heterogeneity, reduced levels of compliance, presentation in general medical rather than specialist settings, less monitoring and standardizing of treatments, and cost pressures.

Despite strong supporting evidence, the uptake of evidence-based procedures remains poor. Misunderstandings and misconceptions about ABA abound, and challenges regarding appropriate research methods to evaluate the effectiveness of individualized interventions contribute to disagreements about what counts as evidence [8]. Applied behavior analysis has been extensively recognized as the gold standard for treating ASD. This acknowledgment comes from decades of research and a large body of supporting evidence [7]. Applied behavior analysis is popular and widely preferred. The ranking or placement of therapies for ABA can vary based on several factors, such as the child's individual needs. Other treatments include speech, physical, occupational, nutritional, and cognitive behavioral therapy, play therapy, social skills training, and developmental approaches [9,10].

Original studies 

Peterson et al. [11-13] analyzed and reported their initial and replicative results using large-N designs, with repeated measures analysis delineating the positive impacts of ABA with various samples (n=100, n=98, n=103). They affirmed the ongoing efficacy of ABA using discrete trial training and mass trials within a naturalistic environment with autistic individuals during a series of snapshot studies covering three months, one month, and one month. Since individual behavior and skill progress vary, measurements every two weeks were supported by our board-certified behavioral analysts (BCBAs) and behavioral technicians, who emphasized that gains in two weeks are typical and expected. Given this, upon inspection of the research dataset, we observed progress every two weeks for many individuals. Overall gains were achieved based on our results. All three studies observed statistically significant increases in mean measurements with multiple raters’ composite general target behaviors acquired per session. Numerous comparisons between time points in the initial study [11] and the two replicative studies [12,13] indicated noteworthy upward trends of improvement and statistically significant differences between time points with medium to large effect sizes. Replication increases confidence in the accuracy of the original findings, enhancing our original and second replication's credibility and reliability. Ideally, we feel that more direct replication studies are needed. 

Replication objectives

This third replication’s primary objective is to ascertain the impact of ABA treatment consisting of discrete trial training and mass trials within a naturalistic environment with a sample of 62 autistic individuals covering a five-month snapshot period from August 8, 2023, to January 8, 2024. It is hypothesized that the child cohorts treated with ABA in discrete trial training and mass trials in a naturalistic environment will demonstrate statistically significant progress toward target behavioral goals over the five-month snapshot period. It is also hypothesized that the time variable will significantly interact with age categories to produce significant improvement effects between time within age categories, as demonstrated by an increase in general cumulative target behaviors.

## Materials and methods

Participants and setting 

Retrospective chart review data were collected from a cohort of 62 autistic individuals using the Catalyst tracking software (Catalyst Software Corp., New York City, NY) who were administered ABA treatment over a five-month snapshot period from August 8, 2023, to January 8, 2024, measuring cumulative target behaviors. Data collection was conducted at The Oxford Center in Brighton, MI. Reporting and manuscript preparation adhered to the Strengthening the Reporting of Observational Studies in Epidemiology (STROBE) guidelines.

The sample of autistic individuals exhibited a range of clinical characteristics with varying severity and manifestation. The sample individuals with autism avoid or do not maintain eye contact, may not respond to their names, and may not show typical facial expressions. They may have difficulty playing simple interactive games, using gestures, sharing interests with others, and pointing to show something interesting. They may not notice when others are hurt or upset and may not join other individuals in play. They may have delayed language skills and repeat words or phrases repeatedly. They may have difficulty following directions and identifying stimuli upon request. They may line up toys or objects and get upset when the order changes. They may be focused on parts of objects (for example, chair legs) and have obsessive interests. They may insist on following certain routines. The sample with autism may have unusual reactions to sound, smell, taste, look, or feel. They may have delayed movement skills, hyperactive, impulsive, and inattentive behavior, epilepsy or seizure disorder, unusual eating and sleeping habits, gastrointestinal issues (for example, constipation), distinctive mood or emotional reactions, anxiety, stress, or excessive worry. Note that not all our samples will exhibit all of these behaviors.

Inclusion & exclusion criteria

Inclusion Criteria

Male and female participants were included in the study. Any autistic individual between the ages of one and 73 who was medically cleared for treatment and had an official diagnosis of autism spectrum disorder by a psychiatrist, psychologist, or primary care physician was included.

Exclusion Criteria

The study excluded individuals without a diagnosis of ASD, those with a medical condition or disability that makes ABA therapy unsafe, and individuals with a history of abuse, neglect, or trauma that may interfere with their ability to benefit from ABA therapy. Individuals who received another intervention were incompatible with ABA therapy, and those with families and providers who could not resolve important issues related to the treatment plan were excluded.

Method of data collection 

Behavioral measurements with autistic individuals were gathered by behavioral technicians daily and recorded in the Catalyst behavioral software. The duration of the data collection period was 22 weeks minus one day from August 8, 2023, to January 8, 2024. The authors decided that measurements every two weeks appeared practical regarding the dataset build, considering individual behavior and skill development variability. Our BCBAs and behavioral technicians consistently observed typical and expected gains within this biweekly timeframe. Upon analyzing the research dataset, we noted progress occurring every two weeks for numerous individuals. These consistent positive trends contributed to overall gains, as evidenced by our results. 

Dependent variable

The dependent (outcome) variable was the number of cumulative target mastery behaviors achieved per session, measured at 12 time points, which were as follows: time 1: baseline; time 2: two weeks; time 3: four weeks; time 4: six weeks; time 5: eight weeks; time 6: 10 weeks; time 7: 12 weeks; time 8: 14 weeks; time 9: 16 weeks; time 10: 18 weeks; time 11: 20 weeks; and time 12: 22 weeks. Catalyst is an ABA data tabulation program that produces case notes and behavioral scores for repeated measures and outcome data for discrete trials teaching behavioral targets. Graphs in Catalyst track quantitative progress and lack of progress with targeted behaviors and automatically determine mastered targets as respective criteria are achieved. 

The composite scores (target behaviors achieved) from multiple behavioral raters (the behavior technicians) represented the count of mastered general target behaviors. These scores were recorded at 12 intervals, each two weeks apart, over five months. These “general aggregate target behaviors,” as defined by BCBAs and behavioral technicians at The Oxford Center, encompassed a range of daily living skills [11-13]. These included routines for organization, time management, eating, toileting, and hygiene. Participants were taught expressive communication skills, which involved using words and phrases, expanding their vocalizations to include more complex vocabulary, enhancing conversational skills, greeting others, responding to greetings, asking for help, and making requests [11-13]. Emphasis was also placed on receptive language skills, such as following instructions and identifying requested stimuli. Social skills training was provided, including taking turns, sharing, being assertive, interacting with peers, and responding appropriately to new acquaintances. Community skills were practiced in real-world settings and included interactions with cashiers, making purchases, managing money, grocery shopping, ordering food at restaurants, interacting with law enforcement, walking safely on sidewalks, playing safely in parks, and learning how to interact safely with strangers [11-13].

Experimental design: repeated measures over time 

Repeated measures designs allow researchers to measure how the treatment affects each child on an ongoing basis to assess the empirical effectiveness of treatments more precisely through observation and analysis. Repeated measures designs look at response outcomes measured on the same experimental unit at various times or under different conditions. In repeated measures designs, each subject serves as their own control [14]. 

Applied behavior analysis interventions

Discrete trial training (DTT), an applied behavior analytic approach, simplifies complexity by breaking down large tasks into small, individualized steps. It employs straightforward and systematic methods for teaching these tasks. Within DTT, mass trials involve repeatedly presenting the same stimulus until the learner responds correctly. Naturalistic environment training (NET), another form of ABA, teaches behavioral skills within a natural learning environment. It leverages the learner’s preferences and interests as motivation [11-13]. A blend of DTT, mass trials, and NET can significantly benefit autistic children by enhancing cognitive, language, social, and adaptive skill development. Discrete trial training helps autistic children learn appropriate responses to various situations, improving communication and relationships with family, classmates, and peers. Skills like matching, discrimination, and imitation, taught through DTT, enhance learning that might be challenging to acquire in naturalistic settings [11-13]. Mass trials expedite the acquisition of new behaviors by exposing autistic children to the same or similar stimuli repeatedly. This method strengthens memory and recall abilities, aiding in retaining learned behaviors over time. Naturalistic environment training facilitates the transfer of generalization skills from discrete trial training to different contexts (people, materials, and settings). Using naturally occurring reinforcements, NET enhances motivation, spontaneity, and engagement [11-13].

Inter-observer reliability

A two-way random effects model was computed, where people's effects and impact measures are also arbitrary. We used the interclass correlation coefficient (ICC) (2), which is used when multiple measurements are made from each averaged rater. The ICC (2) value was 0.980 (95% CI: 0.972-0.987), indicating excellent agreement between the raters. This value was more significant than the average Pearson r (0.856), suggesting that the ICC (2) was more sensitive to the variability among raters and measurements. Cronbach’s alpha for the 12 time point variables was r = 0.980, indicating a high internal consistency reliability [15,16].

Power analysis: study size 

A posteriori power analysis was conducted using GPower 3*1 (Heinrich-Heine-Universität Düsseldorf, Düsseldorf, Germany) [17]. The study indicated that a total sample size of n = 27 participants was required to produce a high effect size (.80) for a repeated measures design with (α) = 0.05 using a mixed repeated measures analysis of variance (ANOVA), with a power equal to 0.987. These parameters indicated a high likelihood that this current study, with 62 participants, possessed an acceptable sample size. 

Statistical methods 

IBM SPSS Statistics for Windows, version 29.0 (IBM Corp., Armonk, NY) was used for all descriptive and inferential statistics. The nominal alpha (α) was set at 0.05. If p-values were less than 0.05 (p < 0.05), a null hypothesis was rejected, and statistical significance was inferred. Demographics and baseline characteristics were summarized for all 62 subjects. Summary statistics for categorical variables, gender, and race/ethnicity; for continuous variables, age, time 1 through time 12 (mean and standard deviation, median, and range were generated). 

A mixed repeated measures ANOVA was run to determine the overall statistical significance between the 12 (time 1 to time 12) levels of the independent variable, as well as any interaction effects between the fixed factor (age categories) and the 12 repeated measures time points assessing target behaviors [18]. 

If an overall significant omnibus F statistic was detected (p < 0.05) within the mixed repeated measures ANOVA, a step-down analysis was performed using resampling multiple comparison procedures in the form of bootstrapped paired t-tests (1000 replications). Using bootstrapping with paired t-tests, resampling methods mitigate potential multiplicity, thereby reducing familywise error rate (FEW) likelihoods [19,20]. 

Suppose an overall significant omnibus interaction F statistic is detected (p < 0.05) within the mixed repeated measures ANOVA, a step-down analysis will be performed using interaction contrasts, comparing each between-subject’s factors (age category) with the within-subjects' factors (time) to determine precisely where the significant differences (effects) came about. 

Institutional review board approval

The Oxford Center was issued approval number 1-1703366-1 from the Western Institutional Review Board (WIRB)-Copernicus Group.

## Results

Descriptive statistics 

Demographics 

For the sample of 62 autistic individuals, the age was M=8.65, SD=4.53, the median was eight years, the minimum was two years, and the maximum was 26 years. There were 46 males (74.2%) and 14 females (22.6%), with two (3.2%) missing values. There were 34 Caucasian participants (54.8%), two Asian participants (3.2%), four Hispanic participants (6.5%), 16 Middle Eastern participants (25.8%), and four African American participants. There were two (3.2%) missing values. 

In terms of age categories, nine (14.5%) were in the one- to four-year category, 21 (33.9%) were in the five- to eight-year category, 12 (19.4%) were in the nine- to 12-year category, seven (11.3%) were in the 13- to 16-year category, and two (3.2%) were in the 17- to 26-year category. There were 11 (17.7%) missing values. Two subjects were over 17 years old, e.g., 20 and 26.

Results for descriptive statistics for time 1 through time 12 measurements are illustrated in Table 1.

**Table 1:** Descriptive statistics for repeated measures Data have been represented by n, mean, standard deviation, and median.

Statistics												
	Targets Mastered Time 1: Baseline	Targets Mastered Time 2: 2 Weeks	Targets Mastered Time 3: 4 Weeks	Targets Mastered Time 4: 6 Weeks	Targets Mastered Time 5: 8 Weeks	Targets Mastered Time 6: 10 Weeks	Targets Mastered Time 7: 12 Weeks	Targets Mastered Time 8: 14 Weeks	Targets Mastered Time 9: 16 Weeks	Targets Mastered Time 10: 18 Weeks	Targets Mastered Time 11: 20 Weeks	Targets Mastered Time 12: 22 Weeks
n	62	62	62	62	62	62	62	62	62	62	62	61
Missing	0	0	0	0	0	0	0	0	0	0	0	1
Mean	9.90	12.37	14.24	17.19	22.84	29.53	35.10	38.27	40.42	41.65	42.06	42.61
Median	7	9	12	13.5	18	27	31	35	37	38	38	38
Standard Deviation	11.99	13.77	15.05	16.21	19.80	21.16	24.68	26.41	28.05	29.26	29.83	29.79
Minimum	0	0	0	0	0	0	0	2	2	2	2	2
Maximum	52	55	65	66	83	91	109	119	131	141	141	141

Descriptive statistics for repeated measurements by age groups are presented in Table 2. 

**Table 2:** Descriptive statistics for repeated measurements by age groups The data have been represented by N, mean, standard deviation, and median.

Age Category	Descriptive Statistic	Targets Mastered Time 1: Baseline	Targets Mastered Time 2: 2 Weeks	Targets Mastered Time 3: 4 Weeks	Targets Mastered Time 4: 6 Weeks	Targets Mastered Time 5: 8 Weeks	Targets Mastered Time 6: 10 Weeks	Targets Mastered Time 7: 12 Weeks	Targets Mastered Time 8: 14 Weeks	Targets Mastered Time 9: 16 Weeks	Targets Mastered Time 10: 18 Weeks	Targets Mastered Time 11: 20 Weeks	Targets Mastered Time 12: 22 Weeks		
1 Year to 4 Years	Mean	14.33	17.55	21.11	25.78	33.11	40.78	52.11	56.78	60.00	61.22	62.11	62.11		
	n	9	9	9	9	9	9	9	9	9	9	9	9		
	Standard Deviation	13.59	17.270	19.92	19.82	24.83	24.52	30.48	33.77	35.85	38.23	38.23	38.23		
	Median	9	11	15	28	31	38	50	58	58	58	60	60		
5 Years-8 Years	Mean	9.05	11.33	12.95	14.52	17.52	22.29	26.57	28.86	31.05	32.57	33	33		
	n	21	21	21	21	21	21	21	21	21	21	21	21		
	Standard Deviation	12.24	12.79	13.79	14.46	16.27	18.45	20.49	21.22	22.23	24.51	25.57	25.57		
	Median	5	9	11	11	16	17	22	25	27	27	27	27		
9 Years-12 Years	Mean	14.25	18.5	21.08	24.5	28.92	37.67	47.25	52.5	55.92	57.58	58.17	58.17		
	n	12	12	12	12	12	12	12	12	12	12	12	12		
	Standard Deviation	13.32	15.45	16.32	16.14	21.26	18.93	21.93	25.24	28.27	29.51	30.47	30.47		
	Median	8	11.5	15	16.5	19	37	50	56	57.5	58	58	58		
13 Years-16 Years	Mean	8.57	10.43	11.71	16.28	29.00	33.57	35.43	37.57	38.57	39.86	39.86	44.83		
	n	7	7	7	7	7	7	7	7	7	7	7	6		
	Standard Deviation	10.79	11.94	11.73	19.12	25.02	29.99	31.53	30.08	30.73	29.82	29.82	29.32		
	Median	6	8	14	14	20	20	21	24	24	24	24	34.5		
17 Years-73 Years	Mean	4.5	5.5	6	8.5	16	29.5	31	31.5	31.5	31.5	31.5	31.5		
	n	2	2	2	2	2	2	2	2	2	2	2	2		
	Standard Deviation	4.95	4.95	4.24	0.71	9.89	10.61	12.72	13.44	13.44	13.44	13.44	13.44		
	Median	4.5	5.5	6	8.5	16	29.5	31	31.5	31.5	31.5	31.5	31.5		
Total	Mean	10.97	13.76	15.86	18.86	24.47	31	37.33	40.64	43.06	44.47	44.94	45.64		
	n	51	51	51	51	51	51	51	51	51	51	51	50		
	Standard Deviation	12.31	14.07	15.38	16.64	20.62	21.96	25.66	27.54	29.26	30.61	31.24	31.15		
	Median	8	10	14	14	20	27	33	37	38	38	38	38.5		

Inferential statistics

A mixed repeated measures ANOVA was performed with post hoc tests in the form of paired t-tests with bootstrapping (1,000 replications) and interaction contrasts, beginning with an analysis of the underlying assumptions. The 12 time point measurements were measured at the continuous (ratio/interval) level. The within-subjects factor consists of the same subjects measured at 12 time points. The between-subjects’ factor consists of age category. 

There were four outliers in time 1 (case numbers 26, 33, 43, and 49), three outliers in time 2 (case numbers 26, 33, and 49), three outliers in time 3 (case numbers 26, 33, 55, and 49), one outlier in time 4 (case number 26), one outlier in time 5 (case number 26), zero outliers in time 6, one outlier in times 7, 8, and 9 (case number 26), and two outliers in time 10, 11, and 12 (case numbers 26 and 33). Because of the nature of the learning progress of the population of autistic individuals and this repeated measures analysis, the outliers will be retained as they are natural to the study’s research question. 

The time point variables demonstrated a non-normal configuration. The skewness scores for eight time points were outside the typically accepted range of -1 to +1. Mixed repeated measures ANOVA is quite "robust" for violations of normality, meaning that the assumption can be somewhat violated and still provide valid results [21]. 

Homogeneity of variances for each combination of the within-subjects factor and the between-subjects factor in significance tests is required. “Sphericity” relates to the variances of the differences between the related groups of the within-subject factor for all groups of the between-subjects factor (the within-subjects factor and between-subjects factor) must be approximately equal. 

Mauchly’s test of sphericity in the study indicates that the assumption of sphericity has not been met: Mauchly’s W = 0.000, approximate Chi-Square = 65.351, df = 65, p < 0.001, Greenhouse-Geyser Epsilon = 0.130, Huynh-Feldt Epsilon = 0.145, lower bound = 0.091. 

Therefore, Greenhouse-Geyser Epsilon will be used to adjust the degrees of freedom for the averaged tests of significance. Greenhouse-Geyser Epsilon correction is a common correction statistic used when "sphericity," i.e., homogeneity of variance with every combination of repeated measures timepoints, is not achieved, thus increasing the likelihood of a type I error. It adjusts the degrees of freedom (df), which produces a higher F-critical value, which makes it more difficult to reject the null hypothesis, thus reducing the likelihood of a Type I error. [18]. Greenhouse-Geyser Epsilon-corrected F-values are reported in the Results section. 

Several investigations [18,21] and others using Monte Carlo simulations into the robustness of generalized linear models (GLMs), of which mixed (between x within) ANOVA is a member, have been reported, suggesting robustness (the likelihoods of Type I error are reduced). 

*Mixed Repeated Measures ANOVA: Main Effects* 

There was a significant main effect (sphericity assumed) on the dependent variable (targets mastered) across time, F(11,495) = 55.432, p < 0.001, ES = 0.552, indicating an overall statistically significant effect (increase in targets mastered) detected across the 12 timepoints of the independent variable (time) over five months, with a large effect size as represented by partial eta squared (Table 3). 

**Table 3:** Mixed repeated measures analysis of variance (ANOVA) The data are represented as sources of variation, assumption violation corrections, sums of squares, degrees of freedom (df), mean square, F-statistic, p-value, and effect size estimates (partial eta squared).

Source	Corrections	Type III Sum of Squares	df	Mean Square	F	p-value (2-Tailed)	Partial Eta Squared
Time	Sphericity Assumed	61397.813	11	5581.619	55.432	< 0.001	0.552
	Greenhouse-Geisser	61397.813	1.429	42961.199	55.432	< 0.001	0.552
	Huynh-Feldt	61397.813	1.594	38515.113	55.432	< 0.001	0.552
	Lower-Bound	61397.813	1	61397.813	55.432	< 0.001	0.552
Time * Age Category	Sphericity Assumed	10356.515	44	235.375	2.338	< 0.001	0.172
	Greenhouse-Geisser	10356.515	5.717	1811.662	2.338	0.044	0.172
	Huynh-Feldt	10356.515	6.376	1624.172	2.338	0.038	0.172
	Lower-Bound	10356.515	4	2589.129	2.338	0.07	0.172
Error (Time)	Sphericity Assumed	49842.716	495	100.692			
	Greenhouse-Geisser	49842.716	64.312	775.02			
	Huynh-Feldt	49842.716	71.736	694.812			
	Lower-Bound	49842.716	45	1107.616			

Mixed Repeated Measures ANOVA: Interaction Effects (Time x Age Category) 

There was a significant interaction effect (sphericity assumed) on the dependent variable (targets mastered) across time and age categories (F(44,495) = 2.338, p < 0.001, ES=0.172, indicating a statistically significant interaction effect detected across the 12 timepoints of the independent variable (time) with the age category (Table 3). 

Post Hoc Analyses: Multiple Comparisons

Multiple comparisons using bootstrapped paired t-tests indicated significance (p < 0.05) on time points 1-9 and non-significance (p > 0.05) on time points 9-12 (Table 4).

**Table 4:** Multiple comparisons using bootstrapped paired samples test Results are presented as mean difference, bootstrap bias, standard error, p-value, 95% confidence interval for the mean difference, effect size (Cohen's d), and 95% confidence interval for effect size (Cohen's d).

Bootstrap for Paired Samples Test	Outcome Variable	Mean Difference	Bootstrap Bias	Standard Error	p-value (2-tailed)	95% Confidence Interval	95% Confidence Interval	Effect Size-Cohen's (d)	95% Confidence Interval for (d)	95% Confidence Interval for (d)
NA	NA	NA	NA	NA	NA	Lower	Upper	NA	Lower	Upper
Pair 1	Targets Mastered Time 1: Baseline - Targets Mastered Time 2: 2 Weeks	-2.5082	0.02626	0.5237	<0.001	-3.60656	-1.5082	-0.618	-0.890	-0.342
Pair 2	Targets Mastered Time 2: 2 Weeks - Targets Mastered Time 3: 4 Weeks	-1.88525	0.00866	0.34001	<0.001	-2.57377	-1.2623	-0.702	-0.981	-0.420
Pair 3	Targets Mastered Time 3: 4 Weeks - Targets Mastered Time 4: 6 Weeks	-3.000	-0.01244	0.58756	0.002	-4.24549	-1.96721	-0.641	-0.915	-0.363
Pair 4	Targets Mastered Time 4: 6 Weeks - Targets Mastered Time 5: 8 Weeks	-5.72131	0.02546	0.92043	<0.001	-7.6713	-3.93443	-0.761	-1.044	-0.473
Pair 5	Targets Mastered Time 5: 8 Weeks - Targets Mastered Time 6: 10 Weeks	-6.72131	0.04874	0.77772	<0.001	-8.24549	-5.19672	-1.060	-1.371	-0.743
Pair 6	Targets Mastered Time 6: 10 Weeks - Targets Mastered Time 7: 12 Weeks	-5.65574	-0.0128	0.94881	<0.001	-7.67213	-3.96763	-0.747	-1.029	-0.461
Pair 7	Targets Mastered Time 7: 12 Weeks - Targets Mastered Time 8: 14 Weeks	-3.19672	-0.01718	0.53317	<0.001	-4.27869	-2.22951	-0.725	-1.005	-0.440
Pair 8	Targets Mastered Time 8: 14 Weeks - Targets Mastered Time 9: 16 Weeks	-2.18033	-0.02048	0.49559	<0.001	-3.21311	-1.27869	-0.545	-0.812	-0.274
Pair 9	Targets Mastered Time 9: 16 Weeks - Targets Mastered Time 10: 18 Weeks	-1.2459	0.00721	0.42846	0.057	-2.14754	-0.4918	-0.361	-0.619	-0.100
Pair 10	Targets Mastered Time 10: 18 Weeks - Targets Mastered Time 11: 20 Weeks	-0.42623	0.00466	0.19593	0.099	-0.85246	-0.08197	-0.272	-0.527	-0.016
Pair 11	Targets Mastered Time 11: 20 Weeks - Targets Mastered Time 12: 22 Weeks	-0.01639	-0.00882	0.01218	0.116	-0.04918	-0.01639	-0.128	-0.379	0.124

Time x Age Interaction Contrasts

Interaction contrasts indicated statistically significant differences (p<.05) over time, mostly within the age categories of one to four years, five to eight years, and most of the nine to 12-year age group. There was some significance (p<.05) within the 13- to 16-year-old age group and no significance (p>.05) within the 17- to 26-year-old age groups. Time x age interaction contrasts are presented in Table 5.

**Table 5:** Time x age interaction contrasts *mean difference is significant at the .05 level b. adjustments for multiple comparisons: Bonferroni correction

Interaction Contrasts (Time x Age)	(I) Time	(J) Time	Mean Difference (I-J)	Standard Error	p-value (two-tailed)	95% Confidence Interval for Difference _b_	95% Confidence Interval for Difference _b_
Measure: Targets Mastered						Lower-Bound	Upper-Bound
Age Category							
1 year to 4 years	1	2	-3.222	1.453	1	-8.473	2.028
		3	-6.778*	1.755	0.024	-13.119	-0.436
		4	-11.444*	2.473	0.002	-20.381	-2.508
		5	-18.778*	3.789	< 0.001	-32.469	-5.086
		6	-26.444*	4.337	< 0.001	-42.117	-10.772
		7	-37.778*	5.631	< 0.001	-58.126	-17.43
		8	-42.444*	6.364	< 0.001	-65.442	-19.447
		9	-45.667*	6.868	< 0.001	-70.487	-20.847
		10	-46.889*	7.245	< 0.001	-73.072	-20.705
		11	-47.778*	7.419	< 0.001	-74.588	-20.967
		12	-47.778*	7.419	< 0.001	-74.588	-20.967
	2	1	3.222	1.453	1	-2.028	8.473
		3	-3.556*	0.945	0.032	-6.972	-0.139
		4	-8.222*	1.921	0.006	-15.163	-1.281
		5	-15.556*	3.252	0.001	-27.306	-3.805
		6	-23.222*	3.921	< 0.001	-37.39	-9.054
		7	-34.556*	5.425	< 0.001	-54.162	-14.949
		8	-39.222*	6.232	< 0.001	-61.742	-16.702
		9	-42.444*	6.78	< 0.001	-66.947	-17.942
		10	-43.667*	7.204	< 0.001	-69.7	-17.633
		11	-44.556*	7.401	< 0.001	-71.301	-17.811
		12	-44.556*	7.401	< 0.001	-71.301	-17.811
	3	1	6.778*	1.755	0.024	0.436	13.119
		2	3.556*	0.945	0.032	0.139	6.972
		4	-4.667	1.649	0.458	-10.628	1.294
		5	-12.000*	2.947	0.012	-22.649	-1.351
		6	-19.667*	3.596	< 0.001	-32.662	-6.671
		7	-31.000*	5.069	< 0.001	-49.32	-12.68
		8	-35.667*	5.863	< 0.001	-56.856	-14.477
		9	-38.889*	6.418	< 0.001	-62.083	-15.695
		10	-40.111*	6.812	< 0.001	-64.729	-15.493
		11	-41.000*	7.009	< 0.001	-66.33	-15.67
		12	-41.000*	7.009	< 0.001	-66.33	-15.67
	4	1	11.444*	2.473	0.002	2.508	20.381
		2	8.222*	1.921	0.006	1.281	15.163
		3	4.667	1.649	0.458	-1.294	10.628
		5	-7.333	2.335	0.197	-15.773	1.106
		6	-15.000*	2.995	< 0.001	-25.823	-4.177
		7	-26.333*	4.433	< 0.001	-42.354	-10.313
		8	-31.000*	5.23	< 0.001	-49.899	-12.101
		9	-34.222*	5.844	< 0.001	-55.341	-13.104
		10	-35.444*	6.317	< 0.001	-58.273	-12.616
		11	-36.333*	6.507	< 0.001	-59.849	-12.818
		12	-36.333*	6.507	< 0.001	-59.849	-12.818
	5	1	18.778*	3.789	< 0.001	5.086	32.469
		2	15.556*	3.252	0.001	3.805	27.306
		3	12.000*	2.947	0.012	1.351	22.649
		4	7.333	2.335	0.197	-1.106	15.773
		6	-7.667*	2.021	0.029	-14.97	-0.363
		7	-19.000*	3.529	< 0.001	-31.755	-6.245
		8	-23.667*	4.427	< 0.001	-39.665	-7.669
		9	-26.889*	5.054	< 0.001	-45.153	-8.625
		10	-28.111*	5.545	< 0.001	-48.151	-8.071
		11	-29.000*	5.745	< 0.001	-49.762	-8.238
		12	-29.000*	5.745	< 0.001	-49.762	-8.238
	6	1	26.444*	4.337	< 0.001	10.772	42.117
		2	23.222*	3.921	< 0.001	9.054	37.39
		3	19.667*	3.596	< 0.001	6.671	32.662
		4	15.000*	2.995	< 0.001	4.177	25.823
		5	7.667*	2.021	0.029	0.363	14.97
		7	-11.333*	2.531	0.003	-20.48	-2.187
		8	-16.000*	3.67	0.005	-29.264	-2.736
		9	-19.222*	4.459	0.006	-35.337	-3.108
		10	-20.444*	5.039	0.013	-38.654	-2.235
		11	-21.333*	5.27	0.013	-40.379	-2.287
		12	-21.333*	5.27	0.013	-40.379	-2.287
	7	1	37.778*	5.631	< 0.001	17.43	58.126
		2	34.556*	5.425	< 0.001	14.949	54.162
		3	31.000*	5.069	< 0.001	12.68	49.32
		4	26.333*	4.433	< 0.001	10.313	42.354
		5	19.000*	3.529	< 0.001	6.245	31.755
		6	11.333*	2.531	0.003	2.187	20.48
		8	-4.667	1.52	0.239	-10.16	0.827
		9	-7.889	2.477	0.173	-16.84	1.062
		10	-9.111	3.215	0.452	-20.728	2.506
		11	-10	3.535	0.459	-22.775	2.775
		12	-10	3.535	0.459	-22.775	2.775
	8	1	42.444*	6.364	< 0.001	19.447	65.442
		2	39.222*	6.232	< 0.001	16.702	61.742
		3	35.667*	5.863	< 0.001	14.477	56.856
		4	31.000*	5.23	< 0.001	12.101	49.899
		5	23.667*	4.427	< 0.001	7.669	39.665
		6	16.000*	3.67	0.005	2.736	29.264
		7	4.667	1.52	0.239	-0.827	10.16
		9	-3.222	1.459	1	-8.496	2.052
		10	-4.444	2.333	1	-12.876	3.987
		11	-5.333	2.745	1	-15.255	4.588
		12	-5.333	2.745	1	-15.255	4.588
	9	1	45.667*	6.868	< 0.001	20.847	70.487
		2	42.444*	6.78	< 0.001	17.942	66.947
		3	38.889*	6.418	< 0.001	15.695	62.083
		4	34.222*	5.844	< 0.001	13.104	55.341
		5	26.889*	5.054	< 0.001	8.625	45.153
		6	19.222*	4.459	0.006	3.108	35.337
		7	7.889	2.477	0.173	-1.062	16.84
		8	3.222	1.459	1	-2.052	8.496
		10	-1.222	1.3	1	-5.922	3.477
		11	-2.111	1.705	1	-8.273	4.051
		12	-2.111	1.705	1	-8.273	4.051
	10	1	46.889*	7.245	< 0.001	20.705	73.072
		2	43.667*	7.204	< 0.001	17.633	69.7
		3	40.111*	6.812	< 0.001	15.493	64.729
		4	35.444*	6.317	< 0.001	12.616	58.273
		5	28.111*	5.545	< 0.001	8.071	48.151
		6	20.444*	5.039	0.013	2.235	38.654
		7	9.111	3.215	0.452	-2.506	20.728
		8	4.444	2.333	1	-3.987	12.876
		9	1.222	1.3	1	-3.477	5.922
		11	-0.889	0.586	1	-3.006	1.228
		12	-0.889	0.586	1	-3.006	1.228
	11	1	47.778*	7.419	< 0.001	20.967	74.588
		2	44.556*	7.401	< 0.001	17.811	71.301
		3	41.000*	7.009	< 0.001	15.67	66.33
		4	36.333*	6.507	< 0.001	12.818	59.849
		5	29.000*	5.745	< 0.001	8.238	49.762
		6	21.333*	5.27	0.013	2.287	40.379
		7	10	3.535	0.459	-2.775	22.775
		8	5.333	2.745	1	-4.588	15.255
		9	2.111	1.705	1	-4.051	8.273
		10	0.889	0.586	1	-1.228	3.006
		12	0	0		0	0
	12	1	47.778*	7.419	< 0.001	20.967	74.588
		2	44.556*	7.401	< 0.001	17.811	71.301
		3	41.000*	7.009	< 0.001	15.67	66.33
		4	36.333*	6.507	< 0.001	12.818	59.849
		5	29.000*	5.745	< 0.001	8.238	49.762
		6	21.333*	5.27	0.013	2.287	40.379
		7	10	3.535	0.459	-2.775	22.775
		8	5.333	2.745	1	-4.588	15.255
		9	2.111	1.705	1	-4.051	8.273
		10	0.889	0.586	1	-1.228	3.006
		11	0	0		0	0
5 years - 8 years	1	2	-2.286	0.951	1	-5.723	1.152
		3	-3.905	1.149	0.094	-8.056	0.247
		4	-5.476	1.619	0.099	-11.326	0.374
		5	-8.476	2.48	0.089	-17.439	0.487
		6	-13.238*	2.839	0.002	-23.498	-2.978
		7	-17.524*	3.686	0.001	-30.845	-4.203
		8	-19.810*	4.166	0.001	-34.865	-4.754
		9	-22.000*	4.496	< 0.001	-38.249	-5.751
		10	-23.524*	4.743	< 0.001	-40.665	-6.383
		11	-23.952*	4.857	< 0.001	-41.504	-6.401
		12	-23.952*	4.857	< 0.001	-41.504	-6.401
	2	1	2.286	0.951	1	-1.152	5.723
		3	-1.619	0.619	0.796	-3.855	0.617
		4	-3.19	1.257	0.971	-7.735	1.354
		5	-6.19	2.129	0.372	-13.883	1.502
		6	-10.952*	2.567	0.007	-20.228	-1.677
		7	-15.238*	3.552	0.006	-28.073	-2.403
		8	-17.524*	4.08	0.006	-32.267	-2.781
		9	-19.714*	4.439	0.004	-35.755	-3.674
		10	-21.238*	4.716	0.003	-38.281	-4.195
		11	-21.667*	4.845	0.003	-39.175	-4.158
		12	-21.667*	4.845	0.003	-39.175	-4.158
	3	1	3.905	1.149	0.094	-0.247	8.056
		2	1.619	0.619	0.796	-0.617	3.855
		4	-1.571	1.08	1	-5.474	2.331
		5	-4.571	1.929	1	-11.543	2.4
		6	-9.333*	2.354	0.017	-17.841	-0.826
		7	-13.619*	3.319	0.011	-25.612	-1.626
		8	-15.905*	3.839	0.01	-29.777	-2.033
		9	-18.095*	4.202	0.006	-33.279	-2.911
		10	-19.619*	4.46	0.004	-35.735	-3.503
		11	-20.048*	4.589	0.005	-36.63	-3.465
		12	-20.048*	4.589	0.005	-36.63	-3.465
	4	1	5.476	1.619	0.099	-0.374	11.326
		2	3.19	1.257	0.971	-1.354	7.735
		3	1.571	1.08	1	-2.331	5.474
		5	-3	1.529	1	-8.525	2.525
		6	-7.762*	1.961	0.017	-14.847	-0.677
		7	-12.048*	2.902	0.01	-22.535	-1.56
		8	-14.333*	3.424	0.009	-26.705	-1.961
		9	-16.524*	3.826	0.006	-30.349	-2.698
		10	-18.048*	4.135	0.005	-32.992	-3.103
		11	-18.476*	4.26	0.005	-33.871	-3.082
		12	-18.476*	4.26	0.005	-33.871	-3.082
	5	1	8.476	2.48	0.089	-0.487	17.439
		2	6.19	2.129	0.372	-1.502	13.883
		3	4.571	1.929	1	-2.4	11.543
		4	3	1.529	1	-2.525	8.525
		6	-4.762	1.323	0.052	-9.543	0.019
		7	-9.048*	2.311	0.02	-17.397	-0.698
		8	-11.333*	2.898	0.02	-21.806	-0.86
		9	-13.524*	3.309	0.012	-25.48	-1.567
		10	-15.048*	3.63	0.01	-28.167	-1.928
		11	-15.476*	3.761	0.011	-29.068	-1.884
		12	-15.476*	3.761	0.011	-29.068	-1.884
	6	1	13.238*	2.839	0.002	2.978	23.498
		2	10.952*	2.567	0.007	1.677	20.228
		3	9.333*	2.354	0.017	0.826	17.841
		4	7.762*	1.961	0.017	0.677	14.847
		5	4.762	1.323	0.052	-0.019	9.543
		7	-4.286	1.657	0.858	-10.274	1.702
		8	-6.571	2.403	0.587	-15.255	2.112
		9	-8.762	2.919	0.289	-19.311	1.788
		10	-10.286	3.299	0.209	-22.207	1.635
		11	-10.714	3.45	0.217	-23.183	1.754
		12	-10.714	3.45	0.217	-23.183	1.754
	7	1	17.524*	3.686	0.001	4.203	30.845
		2	15.238*	3.552	0.006	2.403	28.073
		3	13.619*	3.319	0.011	1.626	25.612
		4	12.048*	2.902	0.01	1.56	22.535
		5	9.048*	2.311	0.02	0.698	17.397
		6	4.286	1.657	0.858	-1.702	10.274
		8	-2.286	0.995	1	-5.882	1.311
		9	-4.476	1.621	0.549	-10.336	1.384
		10	-6	2.104	0.433	-13.605	1.605
		11	-6.429	2.314	0.525	-14.792	1.935
		12	-6.429	2.314	0.525	-14.792	1.935
	8	1	19.810*	4.166	0.001	4.754	34.865
		2	17.524*	4.08	0.006	2.781	32.267
		3	15.905*	3.839	0.01	2.033	29.777
		4	14.333*	3.424	0.009	1.961	26.705
		5	11.333*	2.898	0.02	0.86	21.806
		6	6.571	2.403	0.587	-2.112	15.255
		7	2.286	0.995	1	-1.311	5.882
		9	-2.19	0.955	1	-5.643	1.262
		10	-3.714	1.527	1	-9.234	1.806
		11	-4.143	1.797	1	-10.638	2.352
		12	-4.143	1.797	1	-10.638	2.352
	9	1	22.000*	4.496	< .001	5.751	38.249
		2	19.714*	4.439	0.004	3.674	35.755
		3	18.095*	4.202	0.006	2.911	33.279
		4	16.524*	3.826	0.006	2.698	30.349
		5	13.524*	3.309	0.012	1.567	25.48
		6	8.762	2.919	0.289	-1.788	19.311
		7	4.476	1.621	0.549	-1.384	10.336
		8	2.19	0.955	1	-1.262	5.643
		10	-1.524	0.851	1	-4.6	1.553
		11	-1.952	1.116	1	-5.986	2.082
		12	-1.952	1.116	1	-5.986	2.082
	10	1	23.524*	4.743	< .001	6.383	40.665
		2	21.238*	4.716	0.003	4.195	38.281
		3	19.619*	4.46	0.004	3.503	35.735
		4	18.048*	4.135	0.005	3.103	32.992
		5	15.048*	3.63	0.01	1.928	28.167
		6	10.286	3.299	0.209	-1.635	22.207
		7	6	2.104	0.433	-1.605	13.605
		8	3.714	1.527	1	-1.806	9.234
		9	1.524	0.851	1	-1.553	4.6
		11	-0.429	0.383	1	-1.814	0.957
		12	-0.429	0.383	1	-1.814	0.957
	11	1	23.952*	4.857	< 0.001	6.401	41.504
		2	21.667*	4.845	0.003	4.158	39.175
		3	20.048*	4.589	0.005	3.465	36.63
		4	18.476*	4.26	0.005	3.082	33.871
		5	15.476*	3.761	0.011	1.884	29.068
		6	10.714	3.45	0.217	-1.754	23.183
		7	6.429	2.314	0.525	-1.935	14.792
		8	4.143	1.797	1	-2.352	10.638
		9	1.952	1.116	1	-2.082	5.986
		10	0.429	0.383	1	-0.957	1.814
		12	0	0		0	0
	12	1	23.952*	4.857	< 0.001	6.401	41.504
		2	21.667*	4.845	0.003	4.158	39.175
		3	20.048*	4.589	0.005	3.465	36.63
		4	18.476*	4.26	0.005	3.082	33.871
		5	15.476*	3.761	0.011	1.884	29.068
		6	10.714	3.45	0.217	-1.754	23.183
		7	6.429	2.314	0.525	-1.935	14.792
		8	4.143	1.797	1	-2.352	10.638
		9	1.952	1.116	1	-2.082	5.986
		10	0.429	0.383	1	-0.957	1.814
		11	0	0	.	0	0
9 years - 12 years	1	2	-4.25	1.258	0.1	-8.797	0.297
		3	-6.833*	1.52	0.003	-12.325	-1.342
		4	-10.250*	2.142	0.001	-17.989	-2.511
		5	-14.667*	3.281	0.003	-26.524	-2.809
		6	-23.417*	3.756	< 0.001	-36.989	-9.844
		7	-33.000*	4.876	< 0.001	-50.622	-15.378
		8	-38.250*	5.511	< 0.001	-58.166	-18.334
		9	-41.667*	5.948	< 0.001	-63.161	-20.172
		10	-43.333*	6.275	< 0.001	-66.009	-20.658
		11	-43.917*	6.425	< 0.001	-67.135	-20.698
		12	-43.917*	6.425	< 0.001	-67.135	-20.698
	2	1	4.25	1.258	0.1	-0.297	8.797
		3	-2.583	0.819	0.188	-5.542	0.375
		4	-6	1.663	0.051	-12.011	0.011
		5	-10.417*	2.816	0.039	-20.593	-0.24
		6	-19.167*	3.395	< 0.001	-31.437	-6.897
		7	-28.750*	4.698	< 0.001	-45.729	-11.771
		8	-34.000*	5.397	< 0.001	-53.503	-14.497
		9	-37.417*	5.872	< 0.001	-58.637	-16.197
		10	-39.083*	6.239	< 0.001	-61.629	-16.538
		11	-39.667*	6.409	< 0.001	-62.829	-16.505
		12	-39.667*	6.409	< 0.001	-62.829	-16.505
	3	1	6.833*	1.52	0.003	1.342	12.325
		2	2.583	0.819	0.188	-0.375	5.542
		4	-3.417	1.428	1	-8.579	1.746
		5	-7.833	2.552	0.239	-17.056	1.389
		6	-16.583*	3.114	< 0.001	-27.838	-5.329
		7	-26.167*	4.39	< 0.001	-42.032	-10.301
		8	-31.417*	5.078	< 0.001	-49.767	-13.066
		9	-34.833*	5.558	< 0.001	-54.92	-14.747
		10	-36.500*	5.9	< 0.001	-57.82	-15.18
		11	-37.083*	6.07	< 0.001	-59.02	-15.147
		12	-37.083*	6.07	< 0.001	-59.02	-15.147
	4	1	10.250*	2.142	0.001	2.511	17.989
		2	6	1.663	0.051	-0.011	12.011
		3	3.417	1.428	1	-1.746	8.579
		5	-4.417	2.022	1	-11.725	2.892
		6	-13.167*	2.594	< 0.001	-22.539	-3.794
		7	-22.750*	3.839	< 0.001	-36.624	-8.876
		8	-28.000*	4.529	< 0.001	-44.367	-11.633
		9	-31.417*	5.061	< 0.001	-49.706	-13.127
		10	-33.083*	5.471	< 0.001	-52.853	-13.314
		11	-33.667*	5.635	< 0.001	-54.032	-13.302
		12	-33.667*	5.635	< 0.001	-54.032	-13.302
	5	1	14.667*	3.281	0.003	2.809	26.524
		2	10.417*	2.816	0.039	0.24	20.593
		3	7.833	2.552	0.239	-1.389	17.056
		4	4.417	2.022	1	-2.892	11.725
		6	-8.750*	1.75	< 0.001	-15.075	-2.425
		7	-18.333*	3.057	< 0.001	-29.379	-7.288
		8	-23.583*	3.834	< 0.001	-37.438	-9.729
		9	-27.000*	4.377	< 0.001	-42.817	-11.183
		10	-28.667*	4.802	< 0.001	-46.022	-11.311
		11	-29.250*	4.975	< 0.001	-47.23	-11.27
		12	-29.250*	4.975	< 0.001	-47.23	-11.27
	6	1	23.417*	3.756	< 0.001	9.844	36.989
		2	19.167*	3.395	< 0.001	6.897	31.437
		3	16.583*	3.114	< 0.001	5.329	27.838
		4	13.167*	2.594	< 0.001	3.794	22.539
		5	8.750*	1.75	< 0.001	2.425	15.075
		7	-9.583*	2.192	0.005	-17.505	-1.662
		8	-14.833*	3.179	0.002	-26.32	-3.346
		9	-18.250*	3.862	0.002	-32.206	-4.294
		10	-19.917*	4.364	0.003	-35.687	-4.146
		11	-20.500*	4.564	0.003	-36.994	-4.006
		12	-20.500*	4.564	0.003	-36.994	-4.006
	7	1	33.000*	4.876	< 0.001	15.378	50.622
		2	28.750*	4.698	< 0.001	11.771	45.729
		3	26.167*	4.39	< 0.001	10.301	42.032
		4	22.750*	3.839	< 0.001	8.876	36.624
		5	18.333*	3.057	< .001	7.288	29.379
		6	9.583*	2.192	0.005	1.662	17.505
		8	-5.250*	1.316	0.016	-10.007	-0.493
		9	-8.667*	2.145	0.014	-16.418	-0.915
		10	-10.333*	2.784	0.037	-20.394	-0.273
		11	-10.917	3.062	0.058	-21.98	0.147
		12	-10.917	3.062	0.058	-21.98	0.147
	8	1	38.250*	5.511	< 0.001	18.334	58.166
		2	34.000*	5.397	< 0.001	14.497	53.503
		3	31.417*	5.078	< 0.001	13.066	49.767
		4	28.000*	4.529	< 0.001	11.633	44.367
		5	23.583*	3.834	< 0.001	9.729	37.438
		6	14.833*	3.179	0.002	3.346	26.32
		7	5.250*	1.316	0.016	0.493	10.007
		9	-3.417	1.264	0.637	-7.984	1.151
		10	-5.083	2.021	1	-12.385	2.219
		11	-5.667	2.378	1	-14.259	2.925
		12	-5.667	2.378	1	-14.259	2.925
	9	1	41.667*	5.948	< 0.001	20.172	63.161
		2	37.417*	5.872	< 0.001	16.197	58.637
		3	34.833*	5.558	< 0.001	14.747	54.92
		4	31.417*	5.061	< 0.001	13.127	49.706
		5	27.000*	4.377	< 0.001	11.183	42.817
		6	18.250*	3.862	0.002	4.294	32.206
		7	8.667*	2.145	0.014	0.915	16.418
		8	3.417	1.264	0.637	-1.151	7.984
		10	-1.667	1.126	1	-5.737	2.403
		11	-2.25	1.477	1	-7.587	3.087
		12	-2.25	1.477	1	-7.587	3.087
	10	1	43.333*	6.275	< 0.001	20.658	66.009
		2	39.083*	6.239	< 0.001	16.538	61.629
		3	36.500*	5.9	< 0.001	15.18	57.82
		4	33.083*	5.471	< 0.001	13.314	52.853
		5	28.667*	4.802	< 0.001	11.311	46.022
		6	19.917*	4.364	0.003	4.146	35.687
		7	10.333*	2.784	0.037	0.273	20.394
		8	5.083	2.021	1	-2.219	12.385
		9	1.667	1.126	1	-2.403	5.737
		11	-0.583	0.507	1	-2.416	1.25
		12	-0.583	0.507	1	-2.416	1.25
	11	1	43.917*	6.425	< 0.001	20.698	67.135
		2	39.667*	6.409	< 0.001	16.505	62.829
		3	37.083*	6.07	< 0.001	15.147	59.02
		4	33.667*	5.635	< 0.001	13.302	54.032
		5	29.250*	4.975	< 0.001	11.27	47.23
		6	20.500*	4.564	0.003	4.006	36.994
		7	10.917	3.062	0.058	-0.147	21.98
		8	5.667	2.378	1	-2.925	14.259
		9	2.25	1.477	1	-3.087	7.587
		10	0.583	0.507	1	-1.25	2.416
		12	0	0		0	0
	12	1	43.917*	6.425	< 0.001	20.698	67.135
		2	39.667*	6.409	< 0.001	16.505	62.829
		3	37.083*	6.07	< 0.001	15.147	59.02
		4	33.667*	5.635	< 0.001	13.302	54.032
		5	29.250*	4.975	< 0.001	11.27	47.23
		6	20.500*	4.564	0.003	4.006	36.994
		7	10.917	3.062	0.058	-0.147	21.98
		8	5.667	2.378	1	-2.925	14.259
		9	2.25	1.477	1	-3.087	7.587
		10	0.583	0.507	1	-1.25	2.416
		11	0	0		0	0
13 years - 16 years	1	2	-2.167	1.779	1	-8.597	4.264
		3	-3.5	2.149	1	-11.266	4.266
		4	-8.833	3.029	0.363	-19.778	2.111
		5	-23.500*	4.64	< 0.001	-40.269	-6.731
		6	-28.000*	5.311	< 0.001	-47.194	-8.806
		7	-30.167*	6.896	0.005	-55.088	-5.245
		8	-32.333*	7.794	0.01	-60.499	-4.168
		9	-33.500*	8.412	0.016	-63.898	-3.102
		10	-35.000*	8.874	0.018	-67.068	-2.932
		11	-35.000*	9.086	0.024	-67.836	-2.164
		12	-35.000*	9.086	0.024	-67.836	-2.164
	2	1	2.167	1.779	1	-4.264	8.597
		3	-1.333	1.158	1	-5.517	2.851
		4	-6.667	2.352	0.453	-15.168	1.834
		5	-21.333*	3.982	< 0.001	-35.725	-6.941
		6	-25.833*	4.802	< 0.001	-43.186	-8.481
		7	-28.000*	6.645	0.008	-52.012	-3.988
		8	-30.167*	7.632	0.018	-57.748	-2.585
		9	-31.333*	8.304	0.031	-61.343	-1.324
		10	-32.833*	8.823	0.036	-64.717	-0.949
		11	-32.833*	9.064	0.049	-65.589	-0.078
		12	-32.833*	9.064	0.049	-65.589	-0.078
	3	1	3.5	2.149	1	-4.266	11.266
		2	1.333	1.158	1	-2.851	5.517
		4	-5.333	2.02	0.749	-12.634	1.967
		5	-20.000*	3.609	< 0.001	-33.042	-6.958
		6	-24.500*	4.404	< 0.001	-40.417	-8.583
		7	-26.667*	6.209	0.006	-49.104	-4.23
		8	-28.833*	7.181	0.015	-54.785	-2.882
		9	-30.000*	7.861	0.027	-58.407	-1.593
		10	-31.500*	8.343	0.031	-61.651	-1.349
		11	-31.500*	8.585	0.042	-62.523	-0.477
		12	-31.500*	8.585	0.042	-62.523	-0.477
	4	1	8.833	3.029	0.363	-2.111	19.778
		2	6.667	2.352	0.453	-1.834	15.168
		3	5.333	2.02	0.749	-1.967	12.634
		5	-14.667*	2.86	< 0.001	-25.003	-4.331
		6	-19.167*	3.668	< 0.001	-32.422	-5.912
		7	-21.333*	5.429	0.019	-40.954	-1.713
		8	-23.500*	6.405	0.042	-46.646	-0.354
		9	-24.667	7.157	0.082	-50.532	1.198
		10	-26.167	7.737	0.099	-54.125	1.792
		11	-26.167	7.97	0.131	-54.967	2.634
		12	-26.167	7.97	0.131	-54.967	2.634
	5	1	23.500*	4.64	< 0.001	6.731	40.269
		2	21.333*	3.982	< 0.001	6.941	35.725
		3	20.000*	3.609	< 0.001	6.958	33.042
		4	14.667*	2.86	< 0.001	4.331	25.003
		6	-4.5	2.475	1	-13.445	4.445
		7	-6.667	4.323	1	-22.288	8.954
		8	-8.833	5.422	1	-28.427	10.76
		9	-10	6.19	1	-32.369	12.369
		10	-11.5	6.792	1	-36.044	13.044
		11	-11.5	7.036	1	-36.928	13.928
		12	-11.5	7.036	1	-36.928	13.928
	6	1	28.000*	5.311	< 0.001	8.806	47.194
		2	25.833*	4.802	< 0.001	8.481	43.186
		3	24.500*	4.404	< 0.001	8.583	40.417
		4	19.167*	3.668	< 0.001	5.912	32.422
		5	4.5	2.475	1	-4.445	13.445
		7	-2.167	3.1	1	-13.369	9.036
		8	-4.333	4.495	1	-20.579	11.912
		9	-5.5	5.461	1	-25.236	14.236
		10	-7	6.171	1	-29.302	15.302
		11	-7	6.455	1	-30.326	16.326
		12	-7	6.455	1	-30.326	16.326
	7	1	30.167*	6.896	0.005	5.245	55.088
		2	28.000*	6.645	0.008	3.988	52.012
		3	26.667*	6.209	0.006	4.23	49.104
		4	21.333*	5.429	0.019	1.713	40.954
		5	6.667	4.323	1	-8.954	22.288
		6	2.167	3.1	1	-9.036	13.369
		8	-2.167	1.862	1	-8.895	4.561
		9	-3.333	3.034	1	-14.296	7.629
		10	-4.833	3.937	1	-19.061	9.394
		11	-4.833	4.33	1	-20.48	10.813
		12	-4.833	4.33	1	-20.48	10.813
	8	1	32.333*	7.794	0.01	4.168	60.499
		2	30.167*	7.632	0.018	2.585	57.748
		3	28.833*	7.181	0.015	2.882	54.785
		4	23.500*	6.405	0.042	0.354	46.646
		5	8.833	5.422	1	-10.76	28.427
		6	4.333	4.495	1	-11.912	20.579
		7	2.167	1.862	1	-4.561	8.895
		9	-1.167	1.787	1	-7.626	5.292
		10	-2.667	2.858	1	-12.993	7.66
		11	-2.667	3.362	1	-14.818	9.484
		12	-2.667	3.362	1	-14.818	9.484
	9	1	33.500*	8.412	0.016	3.102	63.898
		2	31.333*	8.304	0.031	1.324	61.343
		3	30.000*	7.861	0.027	1.593	58.407
		4	24.667	7.157	0.082	-1.198	50.532
		5	10	6.19	1	-12.369	32.369
		6	5.5	5.461	1	-14.236	25.236
		7	3.333	3.034	1	-7.629	14.296
		8	1.167	1.787	1	-5.292	7.626
		10	-1.5	1.593	1	-7.256	4.256
		11	-1.5	2.088	1	-9.047	6.047
		12	-1.5	2.088	1	-9.047	6.047
	10	1	35.000*	8.874	0.018	2.932	67.068
		2	32.833*	8.823	0.036	0.949	64.717
		3	31.500*	8.343	0.031	1.349	61.651
		4	26.167	7.737	0.099	-1.792	54.125
		5	11.5	6.792	1	-13.044	36.044
		6	7	6.171	1	-15.302	29.302
		7	4.833	3.937	1	-9.394	19.061
		8	2.667	2.858	1	-7.66	12.993
		9	1.5	1.593	1	-4.256	7.256
		11	0	0.717	1	-2.592	2.592
		12	0	0.717	1	-2.592	2.592
	11	1	35.000*	9.086	0.024	2.164	67.836
		2	32.833*	9.064	0.049	0.078	65.589
		3	31.500*	8.585	0.042	0.477	62.523
		4	26.167	7.97	0.131	-2.634	54.967
		5	11.5	7.036	1	-13.928	36.928
		6	7	6.455	1	-16.326	30.326
		7	4.833	4.33	1	-10.813	20.48
		8	2.667	3.362	1	-9.484	14.818
		9	1.5	2.088	1	-6.047	9.047
		10	0	0.717	1	-2.592	2.592
		12	0	0		0	0
	12	1	35.000*	9.086	0.024	2.164	67.836
		2	32.833*	9.064	0.049	0.078	65.589
		3	31.500*	8.585	0.042	0.477	62.523
		4	26.167	7.97	0.131	-2.634	54.967
		5	11.5	7.036	1	-13.928	36.928
		6	7	6.455	1	-16.326	30.326
		7	4.833	4.33	1	-10.813	20.48
		8	2.667	3.362	1	-9.484	14.818
		9	1.5	2.088	1	-6.047	9.047
		10	0	0.717	1	-2.592	2.592
		11	0	0		0	0
17 years - 26 years	1	2	-1	3.082	1	-12.138	10.138
		3	-1.5	3.722	1	-14.952	11.952
		4	-4	5.246	1	-22.957	14.957
		5	-11.5	8.037	1	-40.544	17.544
		6	-25	9.2	0.614	-58.246	8.246
		7	-26.5	11.944	1	-69.665	16.665
		8	-27	13.499	1	-75.784	21.784
		9	-27	14.569	1	-79.651	25.651
		10	-27	15.37	1	-82.544	28.544
		11	-27	15.738	1	-83.874	29.874
		12	-27	15.738	1	-83.874	29.874
	2	1	1	3.082	1	-10.138	12.138
		3	-0.5	2.005	1	-7.747	6.747
		4	-3	4.074	1	-17.724	11.724
		5	-10.5	6.898	1	-35.427	14.427
		6	-24	8.317	0.394	-54.055	6.055
		7	-25.5	11.509	1	-67.091	16.091
		8	-26	13.219	1	-73.772	21.772
		9	-26	14.383	1	-77.978	25.978
		10	-26	15.282	1	-81.225	29.225
		11	-26	15.699	1	-82.735	30.735
		12	-26	15.699	1	-82.735	30.735
	3	1	1.5	3.722	1	-11.952	14.952
		2	0.5	2.005	1	-6.747	7.747
		4	-2.5	3.499	1	-15.145	10.145
		5	-10	6.251	1	-32.59	12.59
		6	-23.5	7.629	0.232	-51.068	4.068
		7	-25	10.754	1	-63.862	13.862
		8	-25.5	12.438	1	-70.45	19.45
		9	-25.5	13.615	1	-74.702	23.702
		10	-25.5	14.451	1	-77.723	26.723
		11	-25.5	14.869	1	-79.234	28.234
		12	-25.5	14.869	1	-79.234	28.234
	4	1	4	5.246	1	-14.957	22.957
		2	3	4.074	1	-11.724	17.724
		3	2.5	3.499	1	-10.145	15.145
		5	-7.5	4.954	1	-25.403	10.403
		6	-21	6.353	0.123	-43.958	1.958
		7	-22.5	9.404	1	-56.484	11.484
		8	-23	11.094	1	-63.09	17.09
		9	-23	12.397	1	-67.799	21.799
		10	-23	13.4	1	-71.426	25.426
		11	-23	13.804	1	-72.884	26.884
		12	-23	13.804	1	-72.884	26.884
	5	1	11.5	8.037	1	-17.544	40.544
		2	10.5	6.898	1	-14.427	35.427
		3	10	6.251	1	-12.59	32.59
		4	7.5	4.954	1	-10.403	25.403
		6	-13.5	4.287	0.192	-28.992	1.992
		7	-15	7.487	1	-42.057	12.057
		8	-15.5	9.391	1	-49.437	18.437
		9	-15.5	10.721	1	-54.244	23.244
		10	-15.5	11.764	1	-58.012	27.012
		11	-15.5	12.187	1	-59.542	28.542
		12	-15.5	12.187	1	-59.542	28.542
	6	1	25	9.2	0.614	-8.246	58.246
		2	24	8.317	0.394	-6.055	54.055
		3	23.5	7.629	0.232	-4.068	51.068
		4	21	6.353	0.123	-1.958	43.958
		5	13.5	4.287	0.192	-1.992	28.992
		7	-1.5	5.369	1	-20.903	17.903
		8	-2	7.786	1	-30.138	26.138
		9	-2	9.459	1	-36.184	32.184
		10	-2	10.689	1	-40.629	36.629
		11	-2	11.18	1	-42.402	38.402
		12	-2	11.18	1	-42.402	38.402
	7	1	26.5	11.944	1	-16.665	69.665
		2	25.5	11.509	1	-16.091	67.091
		3	25	10.754	1	-13.862	63.862
		4	22.5	9.404	1	-11.484	56.484
		5	15	7.487	1	-12.057	42.057
		6	1.5	5.369	1	-17.903	20.903
		8	-0.5	3.225	1	-12.153	11.153
		9	-0.5	5.254	1	-19.488	18.488
		10	-0.5	6.819	1	-25.143	24.143
		11	-0.5	7.499	1	-27.601	26.601
		12	-0.5	7.499	1	-27.601	26.601
	8	1	27	13.499	1	-21.784	75.784
		2	26	13.219	1	-21.772	73.772
		3	25.5	12.438	1	-19.45	70.45
		4	23	11.094	1	-17.09	63.09
		5	15.5	9.391	1	-18.437	49.437
		6	2	7.786	1	-26.138	30.138
		7	0.5	3.225	1	-11.153	12.153
		9	-2.84E-14	3.096	1	-11.188	11.188
		10	-2.84E-14	4.949	1	-17.886	17.886
		11	2.84E-14	5.824	1	-21.046	21.046
		12	2.84E-14	5.824	1	-21.046	21.046
	9	1	27	14.569	1	-25.651	79.651
		2	26	14.383	1	-25.978	77.978
		3	25.5	13.615	1	-23.702	74.702
		4	23	12.397	1	-21.799	67.799
		5	15.5	10.721	1	-23.244	54.244
		6	2	9.459	1	-32.184	36.184
		7	0.5	5.254	1	-18.488	19.488
		8	2.84E-14	3.096	1	-11.188	11.188
		10	0	2.759	1	-9.97	9.97
		11	5.68E-14	3.617	1	-13.072	13.072
		12	5.68E-14	3.617	1	-13.072	13.072
	10	1	27	15.37	1	-28.544	82.544
		2	26	15.282	1	-29.225	81.225
		3	25.5	14.451	1	-26.723	77.723
		4	23	13.4	1	-25.426	71.426
		5	15.5	11.764	1	-27.012	58.012
		6	2	10.689	1	-36.629	40.629
		7	0.5	6.819	1	-24.143	25.143
		8	2.84E-14	4.949	1	-17.886	17.886
		9	0	2.759	1	-9.97	9.97
		11	5.68E-14	1.243	1	-4.49	4.49
		12	5.68E-14	1.243	1	-4.49	4.49
	11	1	27	15.738	1	-29.874	83.874
		2	26	15.699	1	-30.735	82.735
		3	25.5	14.869	1	-28.234	79.234
		4	23	13.804	1	-26.884	72.884
		5	15.5	12.187	1	-28.542	59.542
		6	2	11.18	1	-38.402	42.402
		7	0.5	7.499	1	-26.601	27.601
		8	-2.84E-14	5.824	1	-21.046	21.046
		9	-5.68E-14	3.617	1	-13.072	13.072
		10	-5.68E-14	1.243	1	-4.49	4.49
		12	0	0		0	0
	12	1	27	15.738	1	-29.874	83.874
		2	26	15.699	1	-30.735	82.735
		3	25.5	14.869	1	-28.234	79.234
		4	23	13.804	1	-26.884	72.884
		5	15.5	12.187	1	-28.542	59.542
		6	2	11.18	1	-38.402	42.402
		7	0.5	7.499	1	-26.601	27.601
		8	-2.84E-14	5.824	1	-21.046	21.046
		9	-5.68E-14	3.617	1	-13.072	13.072
		10	-5.68E-14	1.243	1	-4.49	4.49
		11	0	0		0	0

## Discussion

Applied behavior analysis is a therapeutic strategy aimed at teaching skills and managing behaviors, especially in individuals with ASD. The advantages and medical implications of ABA for ASD encompass enhanced communication abilities, diminished challenging behaviors, improved social interaction abilities, increased independence, better academic performance, an extended attention span, enhanced self-esteem, and an improved quality of life.

From a medical perspective, ABA for ASD has developmental outcomes; specifically, interventions based on ABA have demonstrated moderate effects on intellectual functioning and adaptive behavior in individuals with ASD. Behavioral interventions, specifically those based on ABA, can target specific behaviors (e.g., toilet training), and comprehensive interventions based on ABA are characterized by their early start in childhood, high intensity, personalization to meet each child’s individual needs, simultaneous addressing of multiple skills, and the use of various behavior analytic methods.

Health outcomes include improvements observed across outcome measures with the impact of ABA on children and youth with ASD. It’s crucial to note that while ABA is beneficial, it’s also an intensive process that typically demands many hours per week of patient participation. Furthermore, the effectiveness of ABA can vary among individuals, making it essential to customize the therapy to the individual’s needs.

Previous replications highlighting the effect of ABA on ASD

Replications relative to the impacts of ABA on individuals with ASD are heterogeneous in scope. Hillman et al. [22] replicated and extended prior research by examining the acquisition, maintenance, and generalization of DTT performance of adults with ASD who were interested in careers as behavior technicians. 

Nicolosi & Dillenburger [23] reported a systematic literature review of replication studies over 30 years. Their data showed that the high-intensity, ABA-based University of California at Los Angeles-Young Autism Project (UCLA-YAP) model can benefit children on the autism spectrum, particularly regarding their cognitive functioning and adaptive behavior. Their review concluded that, while more research is always welcome, the impact of the UCLA-YAP model on autism interventions is justified by more than 30 years of outcome evidence. 

Nottingham et al. [24] replicated and extended the study by Griffen and Griffen [25] by comparing a condition in which secondary targets were presented during each trial of a session, a condition in which secondary targets were proposed every other trial and a condition in which secondary targets were proposed about every four trials. Within-subject replications were included for both participants. One of the intermittent presentation schedules was associated with the most optimal outcomes in all four comparisons. 

Barbosa et al.'s [26] replication aimed to evaluate, with strict experimental control, the efficiency of instructional video modeling while training parents of children with ASD to implement discrete trial instruction. Three mother-child dyads participated. Their results showed an increase in the performance accuracy of all mothers in the application of discrete trials, with an average duration of four hours. This instructional tool may affect motivation and broadly promote access to training contingencies, unlike the limitations of face-to-face training. However, it is essential to emphasize that this tool only reaches its full function if it is inserted within a broader training program. 

Ferguson et al. [27] replicated and extended previous research on practical, functional assessment with a different group of researchers and in a different setting (i.e., an early intensive behavioral intervention clinic). This study sought to extend previous literature by including additional social validity measures on the open-ended interview, contingency analysis, treatment, and pre-post measures on parental stress. The results were similar to those of previous studies, with an overall reduction in problem behavior and increased functional communicative responses and compliance with demands. 

Conine et al. [28] replicated and extended a study with three school-aged children with ASD using a multiple baseline design across stories. For some participants and some stories, story recall was mastered under less intensive intervention conditions than in the previous study. When it was necessary to implement the complete intervention package, the effects primarily replicated previous research. Improvements in recall were correlated with increases in the correct answers to comprehension questions. These data have important implications for clinicians and educators providing reading and recall interventions to children with ASD. Results also theoretically impact verbal behavior accounts of memory and recall, suggesting several possible avenues for future research. 

Grow et al.'s [29] replication compared two approaches using progressive prompting with two boys with autism. The results showed that the conditional-only method was a more efficient and reliable teaching procedure than the simple conditional method. The results further called into question the practice of teaching simple discriminations to facilitate the acquisition of conditional discriminations. 

Vladescu et al. [30] replicated a study and evaluated tact acquisition in three, six, and 12 stimulus set sizes. The set sizes of three and six stimuli were associated with the most efficient acquisition, whereas the fixed size of 12 stimuli was not. 

Dhadwal et al. [31] replicated and extended research by teaching three children diagnosed with autism spectrum disorder and other developmental disabilities to respond appropriately to false-belief tasks using behavioral intervention strategies conducted in the natural environment with people in their environment. They used a nonconcurrent multiple-baseline across-participants design to evaluate multiple-exemplary training, prompting, and reinforcement for training correct responses with two false-belief tasks: the hide-and-seek task and the M&M task. They also conducted a pretest/posttest of an untrained false-belief task, the Sally-Anne task. All participants learned to pass the hide-and-seek task and the M&M task and improved their performance on the Sally-Anne task during the post-test.

Strand & Eldevik [32] conducted a systematic replication with the same synthesized treatments as the original study with a young child with ASD enrolled in a home-based Early Intensive Behavior Intervention program (EIBI). Outcomes were similar, with a marked reduction in problem behaviors and increased appropriate requests. Their findings suggested that it is possible to conduct this intervention in a home setting, with weekly consultations with parents. Their study shows the utility of the synthesized treatment in an EIBI program in a home setting and how this can contribute to client time and costs. 

Piper et al. [33] replicated procedures involving learners with ASD in that responding in both full-session and spaced-responding differential reinforcement of low rates of behavior (DRL) schedules were low but not eliminated. Their results provided preliminary evidence to suggest that children with ASD are responsive to signals in DRL arrangements, which may set the stage for evaluating signaled DRL arrangements for socially significant response forms. 

Dowdy et al. [34] conducted a systematic replication to evaluate an intervention that did not require escape extinction for increasing compliance with nail cutting. With two adolescents diagnosed with ASD who resisted nail cutting, they assessed the effects of delivering a preferred edible item contingent on compliance with nail cutting. Results indicated that the treatment reduced participants' escape responses and increased their compliance with nail cutting. 

Summary of this replication’s findings

The primary objective of this study was to replicate new data on 12 time points between August 8, 2023, and January 8, 2024, to show the impact of ABA treatments in a retrospective chart review of 62 individuals with ASD treated with ABA over 12 time points covering five months. The statistical results suggested that ABA intervention over 12 time point measurements significantly increased target behaviors. Expressly, the multiple comparisons between each time point indicated an upward trend of improvement and statistically significant differences between time points in time points 1-8 (p < 0.05), with moderate to high effect sizes (-0.545 to -1.06). There were non-significant mean differences in time points 9-12 (p > 0.05) with minor to moderate effect sizes (-0.128 to -0.361).

The secondary objective was to determine whether an association existed between the 12 time points and age categories. We hypothesized that the children receiving ABA therapy would significantly progress toward targeted general behavioral goals. The secondary hypothesis was that time would significantly interact with age categories, thus yielding significant effects between time and age categories, namely improvement in target behaviors as indicated by time point mean differences. This hypothesis was confirmed within the one- to four-year, five-to-eight-year, and most of the nine- to 12-year categories. This hypothesis was partially confirmed as there was some significance within the 13-16 year age group and not established as there was no significance within the 17-26 year age group. 

Comparison with original studies

This study's results resembled those of Peterson et al. [11-13], with statistically significant findings on the impact of ABA treatments in a five-month snapshot of 62 autistic individuals. Like the first three studies, functional analysis, which consisted of discrete trial training and mass trials within a naturalistic environment, was utilized within a natural environment. Unlike the second study [12], we found a statistically significant interaction (time x age) within many age groups, as mentioned above.

Implications

Our current research presents evidence that may increase confidence in the results of the first three studies [11-13]. The multimodality of discrete trial training and mass trials within a naturalistic environment with autistic children enhances the development of cognitive, language, social, and adaptive skills. The steady increase in this replication study with general target mastery behaviors over the designated 12 time points, covering five months, is noteworthy. Ongoing studies of general ABA broad effectiveness, with large-N studies, can lead to studies to further improve quality and service and support evidence-based practices and improvement [35].

Limitations

There are limitations to this replication. A convenience sample was used and could not be generalized to any larger population. With non-random (convenience) samples such as the one analyzed in this study, there is no ability to generalize results to more extensive circumstances (the population). With random samples, however, whereby every member of the population has an equal likelihood of being selected for the research, this type of sample is as representative of the population (theoretically, anyway) as it can be. Why? Because every member of the population had an equal likelihood of being selected, the likelihood of numerous confounding variables biasing our results is reduced, and we can generalize our sample results back to the population from which the sample was selected. Such is not the case with this sample. Furthermore, given the nature of this multimodal approach, it was impossible to determine statistically significant differences between the groups relative to discrete trial training, mass trials, and naturalistic environment training.

Peterson et al. [11-13] emphasized limitations regarding the seven threats to internal validity, which are always potential sources of bias in repeated measures analyses. Regarding the impact of history, extraneous variables may not be part of the study, or any external events that may have affected outcomes. Maturation involves age-related bodily changes and includes age-related physical changes that can occur with time, such as hunger, tiredness, fatigue, wound healing, surgery recovery, disease progression, etc. Testing relates to the notion that the test may affect the individuals' responses when tested again. These are less of an issue when the tests are routine. Instrumentation refers to any change in measurement ability, including that of any judge, rater, etc. Statistical regression is the tendency for individuals who score extremely high or low on a measure to score closer to the mean of that variable the next time they are measured on it. Selection refers to the potential bias in selecting participants who will serve in the experimental and control groups. Mortality refers to the differential loss of study participants, drop-out rate, or attrition [36].

This is a mixed repeated measures analysis using a within-subjects design. The subjects served as their own control. No “control group” was used as ethical issues precluded the withdrawal of treatment intervention for the research subjects. There is a need in the literature to analyze discrete trial and naturalistic environment training with repeated measures using large-N designs that call for future studies [11-13,35].

## Conclusions

This replicative study puts forth further evidence for the ongoing impact of ABA using discrete trial training and mass trials within a naturalistic environment with autistic individuals during a five-month snapshot. Statistically significant mean differences in target behaviors were determined across the 12 time points, and there were statistically significant associations between many time and age categories. Replicative efficacy studies are common and vital for reporting empirical evidence gathered, helping to confirm the reliability of findings. The results indicate a requirement for further research to explore the intricate impacts of ABA on different developmental benchmarks. This could offer insights for tailoring intervention approaches for individuals with autism. We recommend further replicative studies on ABA and ASD to enhance scientific plausibility.
